# Epstein-Barr Virus-Encoded RNAs: Key Molecules in Viral Pathogenesis

**DOI:** 10.3390/cancers6031615

**Published:** 2014-08-06

**Authors:** Dai Iwakiri

**Affiliations:** Institute for Genetic Medicine, Hokkaido University, N15 W7 Kita-Ku, Sapporo 060-0815, Japan; E-Mail: iwakiri@igm.hokudai.ac.jp; Tel.: +81-11-706-5072

**Keywords:** Epstein-Barr virus, EBER, oncogenesis, innate immunity

## Abstract

The Epstein-Barr virus (EBV) is known as an oncogenic herpesvirus that has been implicated in the pathogenesis of various malignancies. EBV-encoded RNAs (EBERs) are non-coding RNAs expressed abundantly in latently EBV-infected cells. Herein, I summarize the current understanding of the functions of EBERs, including the interactions with cellular factors through which EBERs contribute to EBV-mediated pathogenesis. Previous studies have demonstrated that EBERs are responsible for malignant phenotypes in lymphoid cells, and can induce several cytokines that can promote the growth of various EBV-infected cancer cells. EBERs were also found to bind retinoic acid-inducible gene I (RIG-I) and thus activate its downstream signaling. Furthermore, EBERs induce interleukin-10, an autocrine growth factor for Burkitt’s lymphoma cells, by activating RIG-I/interferon regulatory factor 3 pathway, suggesting that EBER-mediated innate immune signaling modulation contributes to EBV-mediated oncogenesis. Recently, EBV-infected cells were reported to secret EBERs, which were then recognized by toll-like receptor 3 (TLR3), leading to the induction of type I interferon and inflammatory cytokines, and subsequent immune activation. Furthermore, EBER1 was detected in the sera of patients with active EBV-infectious diseases, suggesting that EBER1-meidated TLR3 signaling activation could account for the pathogenesis of active EBV-infectious diseases.

## 1. Introduction

Epstein-Barr virus (EBV), a ubiquitous human herpes virus that has infected more than 90% of the global populations, is associated with a variety of malignancies, including endemic Burkitt’s lymphoma (BL), Hodgkin’s lymphoma, nasopharyngeal carcinoma (NPC), gastric carcinoma (GC), and opportunistic lymphoma. These “EBV-associated” cancers are characterized by the proliferation of monoclonal EBV-infected cells with restricted latent viral gene expression [1].

EBV encodes small nonpolyadenylated, non-coding (nc) RNAs called EBV-encoded RNA (EBER) [1,2]; these are the most abundant viral transcripts in latently EBV-infected cells [3]. EBER1 and EBER2, which are respectively 167 and 172 nucleotides long, are transcribed by RNA polymerase III (pol III) [4]. EBERs can be used as target molecules for the *in situ* hybridization (ISH) detection of EBV-infected cells in tissues [5] and are considered a reliable marker of the presence of EBV, as they are abundantly expressed in latently EBV-infected cells. Previous studies have demonstrated the roles of EBERs in EBV oncogenicity. Expression of EBER in B lymphocytes induces colony formation in soft agar and tumorigenesis in nude mice [6,7]. In addition, EBERs confer resistance to RNA-dependent protein kinase (PKR)-induced apoptosis upon BL cells [8]. Previous studies have also demonstrated that EBERs induce the transcription of various cytokines depending on cell type, such as interleukin-10 (IL-10) in BL cells, insulin-like growth factor-1 (IGF1) in epithelial cells and IL-9 in T cells; these cytokines subsequently act as autocrine growth factors for the EBV-infected cancer cells [9,10,11,12]. More recent studies have shown that EBER1 is sufficient to elicit these phenotypes [13,14]. Regarding the molecular action mechanisms of EBREs, these ncRNAs were reported to contribute to the EBV infection pathogenesis by modulating of innate immune signals [15,16,17].

## 2. Structure of EBERs

**Figure 1:**
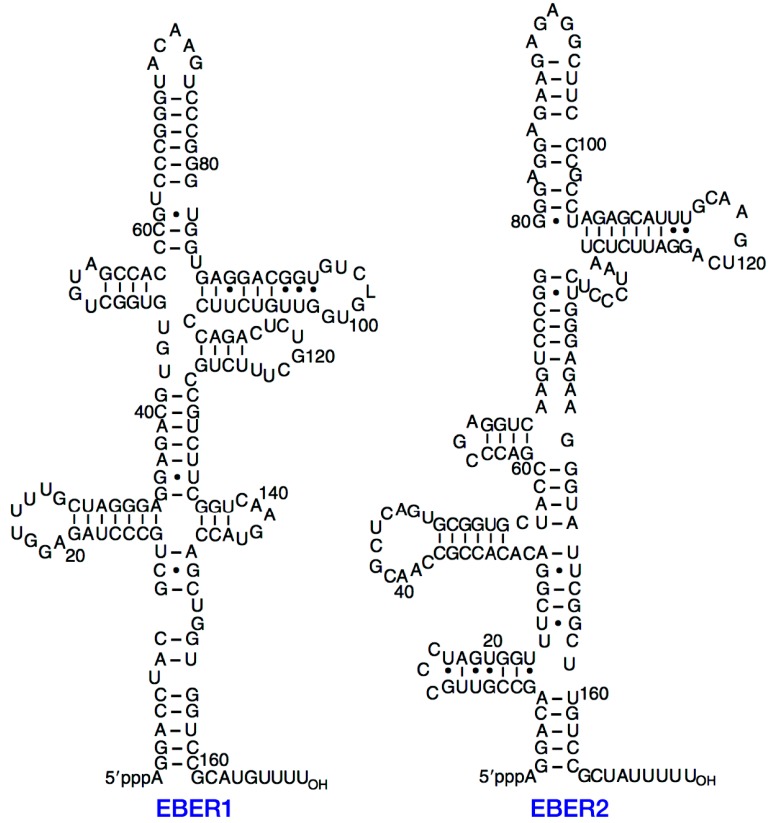
Secondary structures of EBERs. Reproduced from Rosa *et al.* [4]. EBER, Epstein-Barr virus-encoded RNA.

EBER1 and EBER2 are 166 and 172 nucleotides long, respectively [4], and only 54% sequence homology exists between these two RNAs. The EBER genes are separated by 161 base pairs and are transcribed from left to right on the EBV map. Both EBER genes carry intragenic transcription control regions for RNA polymerase (pol) III, and can be transcribed by this molecule. Despite the low (54%) primary sequence similarity, both EBER1 and EBER2 exhibit striking similarities in their secondary structures and feature extensively base-paired structures with several short stem loops (Figure 1, [4]). The secondary structure of EBERs is similar to that of adenovirus-associated RNAs (VAs), which are also small non-polyadenylated RNAs. In addition, the function of VA1 can be partially replaced by EBERs [4]. It has also been reported the similarity between the primary RNA structure of EBER1 and herpesvirus papio type 1 [18]. The differences in the secondary structures suggest that EBER1 and EBER2 have potentially distinct functions, although secondary structure conservation seems to be crucial for the functions of EBERs. The replacement of guanine residues with inosine in EBER1 was reported to disrupt the secondary structure of EBER1, leading to lost ability to form complexes with PKR [19].

The primary sequences of EBERs are highly conserved among a number of EBV strains, with only two reported changes within EBER2 and none in EBER1. A total of eight changes have been observed outside of the coding regions [19]. Family 1 and family 2 parallel type A and type B EBVs (also called type I and type II EBVs) are defined by variations in EBV-nuclear antigen 2 (EBNA2) and EBNA3; however, some isolates appear to be intertypic recombinants, which are often observed in isolates from HIV patients [20]. The high level of sequence conservation among EBERs suggests the importance of EBERs in the virus life cycle.

## 3. Synthesis and Expression of EBERs

EBERs are primarily transcribed by RNA pol III [3]. Class III promoters are characterized by their intragenic location and, in the case of EBERs, have sequences that are nearly identical to the consensus sequences derived from boxes A and B. In addition, EBERs contain three upstream elements that together stimulated *in vivo* expression 50-fold and contain a TATA box and ATF- and Sp1-like promoter elements, which resemble sites associated with typical class II promoters [21], thus indicating the possibility that EBERs can also be transcribed by RNA pol II [21,22].

The EBER copy number appears to be related to the DNA molecule copy number in each cell type [23]. Following the EBV infection of primary B lymphocytes, EBV-determined nuclear antigen 2 (EBNA2) appears first at 6 h post infection, followed by other viral genes, latent membrane proteins (LMPs) and EBERs. On the other hand, the non-transforming P3HR-1 strain, from which the EBNA2 gene is deleted, expresses only the EBNA-leader protein (EBNA-LP) and trace amounts of EBER1 in primary B lymphocytes, whereas the same virus can express EBNA1, EBNA3, EBNA-LP and EBERs in EBV-genome-negative BL cell lines [24]. These findings suggest that EBER expression depends on the host cell, likely through products specific to the cell cycle or the state of B-cell differentiation.

EBER expression appears to be affected by the state of viral life cycle status. A previous study demonstrated downregulated EBER transcription during the switch from latent infection to lytic viral replication [25]. In contrast, the amounts of EBERs remain unaltered within 72 h after the induction of lytic replication. Although both EBERs are transcribed at approximately equal rates, EBER1 has been reported to be present at 10-fold higher levels relative to EBER2, presumably because of the longer half-life of EBER1. In the presence of actinomycin D, the half-lives of EBER1 and EBER2 are 8 to 9 h and 45 min, respectively [26].

EBERs are not expressed in the tissues of oral hairy leukoplakia, Sjögren’s syndrome, salivary gland lymphoma or oral papilloma, in which EBV actively replicates [27,28,29,30] expressed in latently infected cells. Therefore, an ISH targeted EBER1 has been extensively used as a reliable marker of EBV infection in tissue specimens [5]. A previous study reported the heterogeneous EBER expression in individual NPC tumor cells; specifically, some tumor cells expressed high levels of EBER whereas adjacent tumor cells expressed very little or no EBER [31], although it was unclear whether EBER-negative cells were positive for the EBV genome. 

## 4. Localization of EBERs

Although EBERs are located primarily in the nucleus, as was demonstrated via intense nuclear EBER ISH staining [5,32], high-resolution ISH using confocal laser scanning microscopy has demonstrated that EBERs are found in both the cytoplasm and nuclei of interphase cells [33], showing the close association of EBER with the perinuclear region corresponding to the location of the rough endoplasmic reticulum (ER) and Golgi apparatus [33]. More recently, EBERs were shown to be confined to the nucleus [34]. On the other hand, Iwakiri *et al.* [17] reported a new finding that suggested that EBERs are positively secreted in a complex with the lupus antigen (La) protein. Given that EBERs can bind various proteins that are not only restricted to the nucleus but are also located in the cytoplasm, EBERs would be able to localize in the cytoplasm [15,35,36,37].

## 5. Interactions with Cellular Proteins

### 5.1. La Protein

EBERs exist in nuclear ribonucleoprotein (RNP) complexes that can be precipitated using anti-La antibodies, which are associated with systemic lupus erythematosus (SLE) [2]. In these complexes, La binds the oligouridylate stretch at the 3'-termini of all mammalian RNA pol III transcripts; this binding is transient for most RNAs but stable in the case of the EBERs [18]. Although the significance of this interaction is unknown, it is expected to affect the interaction between La and RNA pol III in EBV-infected cells. La is primarily localized in the nucleus; however, it can localize in the cytoplasm under certain condition [37,38,39]. The poliovirus-mediated cleavage of the La nuclear localization signals was reported to result in the shuttling of La from the nucleus to the cytoplasm [39]. A recent study suggested that the EBER-La interaction is significant in terms of the secretion of EBER from EBV-infected cells, as since EBER was found to be mainly released in complex with La [17].

### 5.2. PKR

The adenovirus-associated RNAs, VA1 and VA2, are small RNAs that are transcribed by RNA pol III [40]. Although no striking nucleotide sequence homology exists between EBERs and VAs, similarities exist with respect to size, degree of secondary structure and genomic organization [41]. Similar to VA RNAs, EBERs bind double-stranded RNA-dependent protein kinase (PKR), an interferon (IFN)-inducible serine/threonine kinase that acts as a key mediator of the antiviral effects of IFN [34,42,43,44]. PKR has been reported to bind to the stem-loop IV of EBER1 [45]. IFNs activates PKR, thus leading to phosphorylation of the α-subunit of the protein synthesis initiation factor eIF2 and causing translational inhibition at the level of initiation. *In vitro* assays have demonstrated that EBERs can inhibit PKR activation and block the phosphorylation of eIF2α, thus blocking the eiF2α-mediated inhibition of protein synthesis [19,44,46]. In BL cells, EBERs were also reported to confer resistance to IFN α induced apoptosis by directly binding to PKR and inhibiting its phosphorylation [8]. McKenna *et al.* [47] reported that EBERs/VAs bind preferentially to the latent dephosphorylated form of PKR with a similar affinity to that of dsRNA activators. However, EBERs/VAs prevent PKR dimerization, which is required for efficient PKR trans-autophosphorylation. Consequently, PKR substrate phosphorylation is blocked, allowing protein synthesis to proceed.

### 5.3. L22

A second highly abundant protein designated EBER-associated protein (EAP), was identified in La-containing RNP complexes [48]. EBER1 was reported to primarily bind to EAP [49], and EAP was subsequently shown to be the ribosomal protein L22 [50]. Although its functions are not well understood, L22 was identified as the target of chromosomal translocation in certain leukemia-associated proteins [51,52], suggesting that L22 levels might be a determinant in cell transformation. A previous study suggested that the cellular functions of L22 might involve its association with the human telomerase [53], and L22 has also been shown to interact with a number of small viral RNAs other than EBERs [54,55].

L22 localizes to the nucleoli and cytoplasm in uninfected human B lymphocytes, however, in EBV-infected cells, in which approximately 30%–50% of the L22 has been found to associate with EBER1, L22 relocalizes to the nucleoplasm [50], suggesting that the cellular distribution of L22 in EBV-infected cells results from the L22-EBER1 interaction. A recent study reported that the distribution of L22 was predominately cytoplasmic in EBV-immortalized lymphoblastoid cell lines (LCLs), but independent of EBER1 [56]. Previous studies have shown that EBER1 contains multiple L22-binding domains, including stem-loop III [49], stem-loop IV [57], and stem-loop I [58]. These multiple L22 binding domains suggest the possibility that most EBERs form complexes with L22 *in vivo* and thereby EBERs might modulate protein translation [58]. Elia *et al.* [59] reported that L22 and PKR compete for a common binding site on EBER1. L22 hampers the ability of EBERs to inhibit PKR activation by dsRNA through this competition. Transient EBER1 expression in murine embryonic fibroblasts results in reporter gene upregulation and partially blocks the inhibitory effects of PKR. However, EBER1 is also stimulatory when transfected into PKR-null cells, suggesting a PKR-independent manner of EBER function. L22 expression prevents both the PKR-dependent and -independent effects of EBER1 *in vivo*. These findings suggest that the L22-EBER1 interaction can reduce the biological effect of viral ncRNA, including PKR inhibition and another mechanism by which EBER1 induces gene expression.

### 5.4. Other Interacting Proteins

Recent studies have reported the other cellular proteins that interact with EBERs. The host evokes innate immune responses that eliminate invading pathogens by detecting the presence of infection. Cells express a limited number of germ line-encoded pattern-recognition receptors (PRR) that specifically recognize pathogen-associated molecular patterns within microbes. The retinoic acid-inducible gene I (RIG-I) like receptor (RLR) family, which includes RIG-I [60], melanoma differentiation-associated gene (Mda)-5 [61], and LGP2 [62], comprises cytoplasmic proteins that recognize viral RNA. RLRs are known to play as a key role in IFN-inducible antiviral effects [63]. When RIG-I is activated via an interaction with viral dsRNA, it initiates signaling pathways that lead to the induction of protective cellular genes, including type I IFNs and inflammatory cytokines. RIG-I contains a C-terminal DExD/H-box RNA helicase domain and an N-terminal caspase recruitment domain (CARD). The helicase domain is responsible for dsRNA recognition, and the CARD domain activates downstream signaling cascades via the mitochondrial adaptor IFN-β promoter stimulator (IPS)-1, leading to activating the transcription factors, nuclear factor (NF)-κB and interferon regulatory factor 3 (IRF3) [61,64]. RIG-I is known to recognize 5'-triphosphate RNAs [65], and therefore the EBERs as 5'-triphosphate RNA molecules could also interact with RIG-I [15]. A recent study suggested that EBER-mediated RIG-I activation contributes to EBV oncogenesis [15,16], see below for further details.

The AU-rich element binding factor 1 (AUF1) has the ability to bind to AU-rich elements present in the 3'-untranslated regions of precursor RNA [66]. The association between AUF1 and pre-mRNAs in the nucleus was reported to influence pre-mRNA processing, metabolism, and transport [67], whereas AUF1 alone might play a role in stabilizing certain transcripts [68]. A recent study reported that AUF1 is a novel EBER1 binding protein. The EBER1/AUF1 interaction prevents AUF1 from binding to short-lived mRNAs [36]. How this interaction might affect the EBV-mediated regulation of various genes remains to be clarified.

## 6. Oncogenic Roles of EBERs

EBERs have been linked to the malignant phenotypes of BL cells. The transfection of EBER genes into EBV-negative BL-derived Akata cells restored the capacity for cell growth in soft agar, tumor formation in severe combined immunodeficiency mice, resistance to apoptotic inducers, and the upregulation of bcl-2 that could protect Akata cells from c-Myc induced apoptosis [6,7,69,70]. A recent study of transgenic mice that expressed EBERs in the lymphoid compartment demonstrated the development of lymphoid hyperplasia and, in some cases, lymphoma [14].

Previous studies have demonstrated that EBERs can induce the expression of various cytokines. EBERs induce human IL-10 expression in BL cells [9]; moreover, EBV-positive BL biopsies consistently expressed IL-10, whereas EBV-negative BL biopsies did not. Further analysis revealed that IL-10 acts as an autocrine growth factor for BL cells, suggesting that EBERs contribute to BL development via IL-10 induction [9]. Additionally, EBERs were reported to be responsible for the induction of IL-9, which acts as an autocrine growth factor for T cell proliferation, suggesting that EBERs affect the development of EBV-associated T cell lymphoma [12]. On the other hand, EBERs have been shown to promote the growth and proliferation of epithelial cell lines derived from NPC and GC [10,11,72,73,74]. Iwakiri *et al.* [10,11] demonstrated that EBERs induce IGF1, which acts as an autocrine growth factor for NPC and GC cells. Further, high levels of IGF1 expression were observed in EBV-positive but not in EBV -negative NPC or GC biopsies, suggesting that EBERs contribute to epithelial carcinogenesis via the induction of IGF1 expression [10,11]. A more recent study also demonstrated EBER-mediated IGF1 induction [56].

Regarding the role of EBERs in the process of EBV-induced B cell transformation, an early study reported that EBERs were not essential for primary infection, viral replication, or the B lymphocytes transformation [75]. Other study reported that the 50% transforming dose of an EBER-deleted virus was approximately 100-fold less than that of the EBER-positive EBV; this difference was due to the significantly reduced growth potential of LCLs established with an EBER-deleted virus [76]. Subsequently, EBER2 was found to contribute to the efficient growth transformation of B lymphocytes via the induction of IL-6, which is required for LCL growth [77]. More recently, EBER deletion was reported to have no effect on LCL transformation efficiency or growth [56]. This discrepancy might be due to differences in the EBV strain backgrounds used in these two experiments.

## 7. EBERs-Mediated Pathogenesis via Modulation of Innate Immune Signals

**Figure 2:**
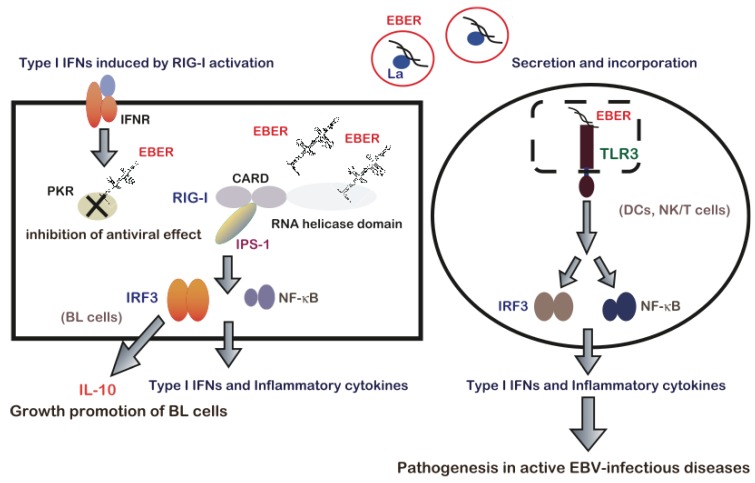
EBER-mediated modulation of innate immune signaling contributes to EBV-mediated pathogenesis. Reproduced from Iwakiri *et al.* [71]. Left, in BL cells, EBERs are recognized by RIG-I via the RNA helicase domain of RIG-I and following recognition, RIG-I associates with the adaptor IPS-1 via its CARD. IPS-1 initiates signaling leading to the activation of IRF3 and NF-κB to induce type I IFNs and inflammatory cytokine expression. EBERs induce the expression of the growth-promoting cytokine IL-10 via RIG-I-mediated IRF3 activation and might support BL development. EBERs also bind to IFN-inducible PKR and block its activity, which is required for the IFN-mediated antiviral effect; therefore, EBV might maintain a latent infection state. Right, activation of innate immunity via TLR3 signaling in response to secreted EBER. During an active EBV-infection, EBER1 is released from EBV-infected lymphocytes primarily in a complex with La. Circulating EBER induces DC maturation via TLR3 signaling and induces type I IFN and inflammatory cytokine production by activating IRF3 and NF-κB. DC activation leads to T cell activation and systemic cytokine release. Furthermore, TLR3-expressing T and NK cells including EBV-infected T or NK cells could be activated by EBER1 through TLR3, thus leading to inflammatory cytokine production. Therefore, immunopathologic diseases caused by active EBV infections including T or NK cell activaiton and hypercytokinemia, could be attributed to EBER1-induced TLR3-mediated T cell activation and cytokinemia. EBER, Epstein-Barr virus encoded RNA; EBV, Epstein-Barr virus; BL, Burkitt’s lymphoma; IPS-1, interferon-β promoter stimulator-1; CARD, caspase recruitment domain; RIG-I, retinoic acid-inducible gene I; DC, dendritic cell; IFN, interferon; NK cell, natural killer cell; IL, interleukin; TLR, Toll-like receptor; PKR, RNA-dependent protein kinase; IRF 3, interferon regulatory factor 3.

Interactions between EBERs and host dsRNA sensors have been reported to play a significant role in EBV-mediated pathogenesis (Figure 2). Samanta *et al.* [15] reported that EBER, which is expected to form dsRNA structures, activates RIG-mediated signaling. The results suggest that in BL cells, RIG-I is constitutively activated by EBERs, leading to the activation of NF-κB and IRF-3, and subsequent induction of type-I IFN. Although IFN induction appears to be disadvantageous for the virus, EBV can maintain a latent infection state because of resistance to IFN, such as that provided by EBER-mediated PKR inhibition [8]. Furthermore, a subsequent study demonstrated the significant role EBER-induced RIG-I activation in oncogenesis. EBER promotes the BL cell growth by inducing expression of anti-inflammatory and growth-promoting cytokine IL-10, in a manner dependent on RIG-I-mediated IRF3 signaling but independent of NF-κB [16].

Toll-like receptors (TLRs) comprise a distinct family of PRRs that sense virus-derived nucleic acids and trigger antiviral innate immune responses by activating signaling cascades via Toll/IL-1 receptor (TIR) domain-containing adaptors [78]. The role of TLR3 in the dsRNA recognition was demonstrated in a study of TLR3-deficient mice [79]. dsRNA-induced signal transduction via TLR3 leads to the recruitment of TIR domain-containing adaptor inducing IFN-β (TRIF) and the subsequent phosphorylation of downstream molecules such as IRF3 and NF-κB [63]. Iwakiri *et al.* [17] demonstrated that EBERs are released extracellular environment and are recognized by TLR3, leading to the induction of type I IFN and inflammatory cytokines. The majority of the released EBER1 exists as a complex with La, suggesting that EBER1 is released from the cells via the active secretion of La. EBV has been known to cause active infectious diseases such as infectious mononucleosis (IM), chronic active EBV infection (CAEBV) and EBV-associated hemophagocytic lymphohistiocytosis (EBV-HLH). IM is characterized by the expansion of reactive T-cells and is most likely an immunopathologic disease, in which the general symptoms are caused by inflammatory cytokines [1]. CAEBV and EBV-HLH are also active EBV infections with persistent or recurrent IM-like symptoms. EBV-HLH is characterized by an EBV infection in CD4-positive T cells or naural killer (NK) cells and the systemic release of inflammatory cytokines, leading to blood cell hemophagocytosis via the activation of macrophages [1,80,81]. On the other hand, in CAEBV, CD8^+^-T cells are primary EBV infection targets [81]. Iwakiri *et al.* [17] demonstrated that sera from patients with IM, CAEBV and EBV-HLH contained EBER1. A further analysis revealed that serum EBER1 activates TLR3 signaling in immune cells, including dendritic cells, suggesting that EBER1, which is released from EBV-infected cells is responsible for EBV-mediated immune activation, and induction of type I IFN and inflammatory cytokines [17]. Because CD8^+^-T cells and NK cells express TLR3 and can be activated by TLR3 signaling [82,83], TLR3-expressing T and NK cells could potentially be activated by EBER1 through TLR3 to produce inflammatory cytokines. Therefore, EBER1-induced activation of innate immunity would account for the immunopathologic diseases caused by active EBV infection. A more recent study of a humanized mice model of fetal EBV-infectious disease observed the EBER1 in the serum, thus suggesting that EBER1 contribute to the disease pathology [84]. In summary, EBERs contribute to EBV infection-related pathogenesis, including cancer and active infectious diseases, through interactions with RIG-I and TLR3 [71].

## 8. Conclusions and Future Direction

Recently, the bioactive extracellular small membrane vesicles also known as exosomes were reported to contribute to cancer development. Exosomes has been shown to contain miRNA, DNA, proteins, and even virus particles [85,86]. Additionally, EBV-infected cells were also reported to produce exosomes that contained viral proteins and miRNA [87]. Previous studies have demonstrated that the La protein can be excreted in exosomes, thus supporting the possibility that EBER is also secreted and transferred to neighboring cells via exosomes [88]. The interactions between EBERs and cellular factors are involved in EBV-mediated pathogenesis, including cancer. A further study of the detailed molecular mechanism is required in order to develop a new therapeutic approach that targets these ncRNAs in EBV-associated cancers.
